# An Improved Single Cell Ultrahigh Throughput Screening Method Based on In Vitro Compartmentalization

**DOI:** 10.1371/journal.pone.0089785

**Published:** 2014-02-24

**Authors:** Fuqiang Ma, Yuan Xie, Chen Huang, Yan Feng, Guangyu Yang

**Affiliations:** 1 State Key Laboratory of Microbial Metabolism, School of Life Sciences and Biotechnology, Shanghai Jiao Tong University, Shanghai, China; 2 Key Laboratory for Molecular Enzymology and Engineering of Ministry of Education, Jilin University, Changchun, China; Albert-Ludwigs-University, Germany

## Abstract

High-throughput screening is a key technique in discovery and engineering of enzymes. *In vitro* compartmentalization based fluorescence-activated cell sorting (IVC-FACS) has recently emerged as a powerful tool for ultrahigh-throughput screening of biocatalysts. However, the accuracy of current IVC-FACS assays is severely limited by the wide polydispersity of micro-reactors generated by homogenizing. Here, an improved protocol based on membrane-extrusion technique was reported to generate the micro-reactors in a more uniform manner. This crucial improvement enables ultrahigh-throughput screening of enzymatic activity at a speed of >10^8^ clones/day with an accuracy that could discriminate as low as two-fold differences in enzymatic activity inside the micro-reactors, which is higher than similar IVC-FACS systems ever have reported. The enzymatic reaction in the micro-reactors has very similar kinetic behavior compared to the bulk reaction system and shows wide dynamic range. By using the modified IVC-FACS, *E. coli* cells with esterase activity could be enriched 330-fold from large excesses of background cells through a single round of sorting. The utility of this new IVC-FACS system was further illustrated by the directed evolution of thermophilic esterase AFEST. The catalytic activity of the very efficient esterase was further improved by ∼2-fold, resulting in several improved mutants with *k*_cat_/*K*_M_ values approaching the diffusion-limited efficiency of ∼10^8^ M^−1^s^−1^.

## Introduction

Fluorescence-activated cell sorting (FACS) has recently become one of the most efficient tools for the screening of large enzyme libraries. By utilizing the elegant cell sorting capability of flow cytometry, it can easily screen a library containing >10^8^ clones per day in a quantitative manner [1]. FACS is especially powerful when it is combined with *in vitro* compartmentalization (IVC) system which artificially generates cell-like compartments as reaction chambers for the enzymatic reactions [2]. This so-called *in vitro* compartmentalization based fluorescence-activated cell sorting (IVC-FACS) technique encapsulates single, enzyme-expressing cells and fluorogenic substrate into individual compartments to form micro-reactors, which are analyzed and sorted by flow cytometry after a certain period of enzymatic reaction. IVC-FACS is compatible with almost any fluorogenic assay system and has been successfully applied in screening various kinds of enzymatic activities, including thiolactonase [3], *β*-galactosidase [4], cellulase [5], *β*-glucosidase [6], glucose oxidase [7], cutinase [8], protease [9], and G-type nerve agent hydrolase [10]. However, most current IVC-FACS systems are suffered from large system error, which is mainly caused by the wide polydispersity of droplet-based micro-reactors. Because the concentrations of enzyme, substrate and product inside w/o/w double emulsion droplets are all determined by the volume of inner water phase, the uniformity of droplets has direct influence on the enzymatic activity readout. Nevertheless, the state of art technology for droplet generation is based on homogenizing, which is simple and fast but difficult to control the size of droplets precisely [3]. The diameter of homogenizing droplets can span as much as 20 times, resulting in up to 8000-fold difference in reaction volume and big error in the downstream screening.

With the recent development of microfluidics, w/o/w double emulsion droplets with high uniformity can be generated by the flow-focusing junction of a microcapillary device [11] or a microfluidic chip [12]. However, current microfluidic techniques still can’t fulfill the requirement of IVC-FACS systems for two reasons. Firstly, the diameters of the droplets generated by microfluidics is usually more than 50 µm, which are too large for an efficient sorting of a normal flow cytometer. Secondly, the generation speed of w/o/w double emulsion droplets in microfluidic chips is at least 4 orders of magnitude slower than that of homogenizing method, which would make the generation of droplets become a new bottleneck in the screening process. New method to generate droplets at a high speed but with good uniformity would greatly increase the accuracy of IVC-FACS assays and would be very valuable in the discovery and engineering of new biocatalysts.

Membrane-extrusion is an alternative method for the fabrication of micro droplets. It has been reported that relatively uniform droplets can be generated by membrane-extrusion because of its gentler shear stress [13]. Mastrobattista et al. have employed this system in the encapsulation of *in vitro* transcription and translation reagent [4], but to the best of our knowledge, it has never been used in single cell encapsulation for IVC-FACS. Besides, it is also not clear about the performance of membrane-extrusion compared with the homogenizing method. In this work, we reported a protocol employing membrane-extrusion technique to compartmentalize enzyme-expressing single cells into w/o/w double emulsion droplets. Our protocol has similar droplet generation speed compared with the commonly-used homogenizing protocol but the resulted droplets are much more homogeneous in reaction volume. The utility of this protocol in IVC-FACS was validated by a model screening of the *E. coli* cells displaying esterase out of large excesses of inactive background cells. In addition, a random mutagenesis library of a thermophilic esterase from *Archaeoglobus fulgidus* was screened to further illustrate the efficacy of our method in a directed evolution context.

## Materials and Methods

### Materials

All chemical reagents used were analytical reagent grade or higher quality. The surface-displaying vector pGF101 [14] and the GFPuv-expressing *E. coli* strain were kindly provided by Dr. Zuoming Zhang (Jilin University, China).

### Preparation of w/o Single Emulsion

For emulsion generation, a mini extruder (Avanti Polar Lipids, AL, USA) equipped with two Gastight 1001 syringes (1 mL, Hamilton, NV, USA) and 8-µm-pored Track-Etch polycarbonate membrane (Millipore, USA) was used. After fitted with 8-µm-pored membrane, the mini extruder was prerinsed twice with 0.5 mL oil phase (light mineral oil containing 2.9% (v/v) ABIL EM90). For emulsification, 100 µL of inner water phase (1×PBS buffer, pH7.4) and 400 µL oil phase were loaded into the same syringe, the mixture was forced into the alternate syringe through the extruder, and directly forced back into the original syringe, completing one time of emulsification. The quality of w/o single emulsion was evaluated using a microscope (50i, Nikon, Japan, 40× object) and the droplet diameter was controlled to 3∼5 µm by varying the emulsification times. After generation, the w/o single emulsion was stored on ice.

### Preparation of w/o/w Double Emulsion

To generate w/o/w double emulsion droplets, a new piece of 8-µm-pored membrane was assembled, and the mini extruder was prerinsed twice with 0.5 mL outer water phase (1×PBS buffer, pH7.4 containing 1% (v/v) TritonX-102). For the secondary emulsification, 200 µL of the primary w/o single emulsion and 400 µL of outer water phase were loaded into two different syringes, respectively. The w/o emulsion was forced into the outer water phase through the extruder, and directly forced back into the original syringe to complete one time of extrusion. The formation of double emulsions was evaluated by real-time observation using a microscope until most of the w/o/w double emulsion droplets were in diameter of ∼10 µm and uniform in size. The w/o/w double emulsion was then stored on ice.

### *E. coli* Cell Surface Display of Thermophilic Esterases AFEST and FnE

Two thermophilic esterases, AFEST from *Archaeoglobus fulgidus* and FnE from *Fervidobacterium nodosum*, were chosen as the model enzymes. The plasmid pGF101 harboring N- and C- terminal sequence of ice nucleation protein (INPNC) was used as surface-displaying vector. Construction of AFEST-pGF101 and FnE-pGF101 recombinant plasmids were achieved through homologous recombination by using CloneEZ (Genscript, Nanjing, China). The fragments of target genes and vector were prepared by PCR, respectively, and there were 15-bp homologous flanks both on the 3′ and 5′ terminal of the two fragments. The sequences of primers used are: AFEST upper 5′-GAGGTAAAGCCATGGATGCTTGATATGCCAATCG-3′, AFEST lower 5′-CCAAAACAGAAGCTTCTAGTCGAACACAAGAAG-3′, FnE upper 5′-GAGGTAAAGCCATGGATGTATTATAACAACGGAATACC-3′, FnE lower 5′-CCAAAACAGCTCGAGCTTATTCTCCAAAAAAGTATTTTATCG-3′, pGF101 upper 5′-AAGCTTCTGTTTTGGCGGATGAGAG-3′, pGF101 lower 5′-CCATGGCTTTACCTCTATCCAGTCATCGTC-3′.

The recombinant plasmids were transformed into *E. coli* JM109 strain to display the esterases on the cell surface.

### Enzymatic Reaction in the Double Emulsion Micro-reactors

AFEST was constructed into pET-28a(+) plasmid (Novagen, Inc., Madison, WI, USA) and over expressed in the cytoplasm of *E. coli* BL21-CodonPlus (DE3) (Stratagene, La Jolla, CA, USA). The cells were lysed by ultrasonication, centrifuged and the supernatant was collected as crude enzyme solution. The primary solution was diluted at a 2-fold gradient, obtaining a series of solutions with the activity ratios of 1, 2, 4, 8, 16 and 32, respectively. These enzyme solutions were then used as the inner water phase and encapsulated into w/o/w double emulsions. Fluorogenic substrate fluorescein dibutyrate (Sigma, 10 mM in dimethyl sulfoxide) was added to the outer water phase to the final concentration of 0.5 mM. The mixtures were incubated on a thermo-shaker at 1000 rpm at 37°C for different periods of time and the fluorescence signals of different samples were detected by a flow cytometer.

### Single-cell Enzymatic Reaction in Double Emulsion Micro-reactors

The *E. coli* JM109 strain harboring esterase-pGF101 plasmid was inoculated into LB medium and incubated at 220 rpm at 37°C. When the OD600 of cell culture reached 0.8–1.0, isopropyl-β-D-thiogalactoside (IPTG) was added to the final concentration of 1.0 mM. The incubation temperature was then lowered to 25°C overnight at 220 rpm. Before encapsulation, 1 mL cell culture was collected, washed with PBS twice and then resuspended in 1 mL of PBS. 100 µL of the cell suspension (containing ∼2.5×10^8^ cells) was used as the inner water phase for double emulsion generation. For reaction, 0.5 mM fluorescein dibutyrate was added to the outer water phase. The reaction system was incubated at 1000 rpm on a thermo-shaker at 37°C. After a period of time, the mixture was cooled on ice to terminate the reaction and then detected by a flow cytometer.

### FACS Assays of Double Emulsion Droplets

Flow cytometry analysis and sorting of double emulsion droplets was performed on a BD FACSAria II (Becton-Dickinson, USA) cytometer operated by the BD FACSDava software. The w/o/w double emulsion was diluted 200-fold in the outer water phase and then loaded into flow cytometry with PBS as sheath fluid. The flow cytometry was fitted with a 70 µm nozzle, a near UV laser emitting at 375 nm and a blue laser emitting at 488 nm. The band-pass filters of 530/30 nm and 450/40 nm were used to detect fluorescence signal of the product (fluorescein) and the internal standard (*7-Hydroxycoumarin-3-carboxylic acid*), respectively. The detecting threshold was set at SSC>10,000. The applied analyzing rate was ∼8000 events/s and the sorter was triggered on product fluorescence.

### Model Screening

The *E. coli* cells expressing esterase AFEST on their surface were mixed with cells harboring empty plasmid pGF101 at ratios of 1∶10, 1∶100, and 1∶1000, respectively. The cell-mixtures were encapsulated into w/o/w double emulsions and reacted with fluorescein dibutyrate. After reaction, the samples were sorted by flow cytometry and different percentages of the brightest droplets in fluorescence were collected into 2-mL eppendorf tubes containing 1 mL LB medium. After growing the collected cells on agar plates, negative and positive colonies were identified by their enzymatic activity in 96-well plates using 4-nitrophenyl butyrate as substrate. The enrichment factors were calculated according to the positive ratios before and after sorting.

### Directed Evolution of AFEST

Error-prone PCR (ep-PCR) was performed to generate the mutant libraries. The mutation rates were adjusted by varying the manganese ion concentrations. The ep-PCR system contained: DreamTaq (0.05 U/µL) and its buffer (Takara), dATP (250 µM), dGTP (250 µM), dCTP (1050 µM), dTTP (1050 µM), upper primer for AFEST upper (0.4 µM), lower primer for AFEST lower (0.4 µM), AFEST-pET-28a template (0.2 ng/µL) and manganese chloride (0.2–0.8 mM). The mixture was divided into aliquots of 25 µL for ep-PCR (95°C for 3 min, 1 cycle; 95°C for 15 s/55°C for 30 s/72°C for 1 min, 30 cycles; 72°C for 5 min, 1 cycle). The purified fraction was digested by *Nco*I and *Hind*III, constructed into pGF101 vector using *T4* ligase and transformed into *E. coli* 10G electrocompetent cells (Lucigen, USA). The transformed cells were grown overnight at 37°C in LB medium supplemented with ampicillin (100 µg/mL) and the library plasmid DNA was extracted. Several individual clones from each library were sequenced. The library constructed with 0.4 and 0.6 mM MnCl_2_ were shown to have an average mutational frequency of ∼2 and ∼5 mutations per gene. The plasmid DNA from the two libraries was mixed and then transformed into *E. coli* JM109 for screening.

*E. coli* JM109 strain harboring the mixed library was cultured in LB liquid medium and induced by IPTG. The cells displaying AFEST variants were washed with PBS and encapsulated into w/o/w double emulsion droplets. After reacted with fluorescein dibutyrate (0.5 mM in outer water phase) at 37°C for 15 min, the droplets with the highest fluorescent intensity (approximately 0.1% of cell-containing droplets) were sorted into empty 2-mL eppendorf tubes. The positive genes were recovered by PCR using the sorted cells as PCR template. The recovery-PCR system contained: DreamTaq (0.05 U/µL) and its buffer (Takara), dATP (250 µM), dGTP (250 µM), dCTP (250 µM), dTTP (250 µM), upper primer for AFEST upper (0.4 µM), lower primer for AFEST lower (0.4 µM), sorted cells (∼1000 cells in 5 µL sorted sample). The thermo cycler was programmed at: 95°C for 3 min, 1 cycle; 95°C for 15 s/55°C for 30 s/72°C for 1 min, 30 cycles; 72°C for 5 min, 1 cycle. The PCR product was reconstructed into pET-28a(+) plasmid and transformed into *E. coli* BL21-CodonPlus (DE3) strain. Single clones were picked into 96-well plates containing 200 µL LB medium supplemented with ampicillin (100 µg/mL) and cultivated at 37°C, 400 rpm. After inducing the cells by 1.0 mM of IPTG at 25°C for 20 h, the cells were harvested by centrifuging at 3000 rpm for 30 min and then were lysed by 3 rounds of freeze-thaw treatment. The cell lysate was centrifuged at 3000 rpm for 30 min and 10 µL supernatant was mixed with 10 µL 4-nitrophenyl butyrate (Sigma, 10 mM in acetonitrile) and 180 µL PBS. After incubated at 37°C for 5 min, the activity of each mutant was measured at OD405. Positive mutants with higher activity over wild type AFEST were selected and sequenced. Mutant enzyme was prepared using Ni-NTA column affinity chromatography and the kinetic parameters were studied according to method described before [15].

## Results and Discussion

### W/o/w Double Emulsion Droplet Generation by Membrane-extrusion

Generation of micro-droplets comprises the bottleneck of the accuracy in the entire IVC-FACS process. Ideal micro-droplets generating method for IVC-FACS should be: a) easy to operate, b) having high generation speed to match the requirement of ultrahigh-throughput, c) able to generate size-uniform droplets to ensure the accurate measurement of enzyme activity. The most often used homogenizing method is easy to operate and has very high emulsion generating speed (∼10^10^ droplets in 10 min) [3]. However, droplets made from this method are very heterogeneous in size, which severely hampers the accurate enzymatic activity readout. To improve droplet uniformity, we tried membrane-extrusion method as an alternative for encapsulating single cells. At first, we tried the recipe of oil phase reported by Mastrobattista et al, by which they used for the compartmentalization of the *in vitro* transcription and translation system [4]. However, we found that the surfactant cholesterol had poor solubility in the oil phase decane, requiring the whole emulsification process to be performed at elevated temperature, which greatly reduced the feasibility of the operation. To solve this problem, light mineral oil was chosen as the oil phase and ABIL EM90 was used as the surfactant. By doing so, the emulsification could be conveniently performed at room temperature and the potential hazard of decane could also be avoided.

**Figure 1:**
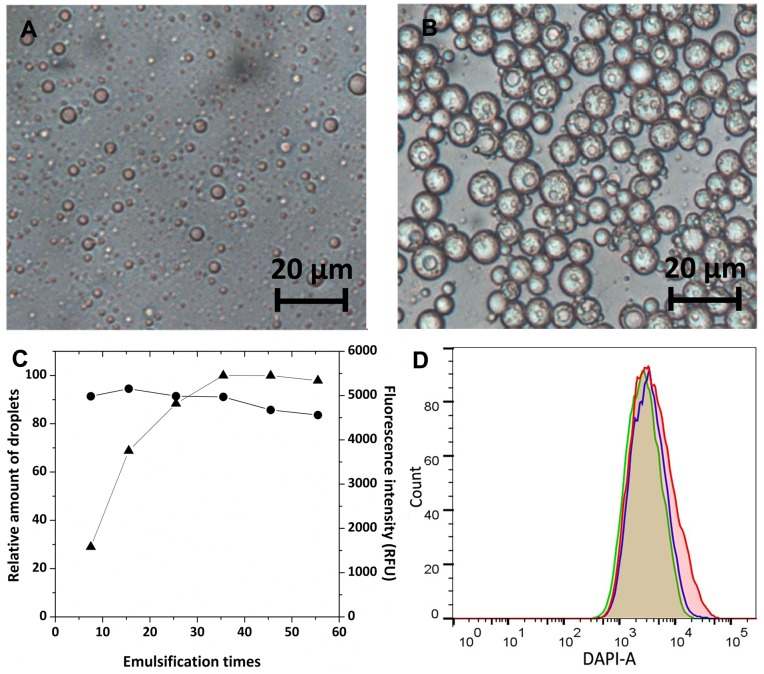
Droplets generated by membrane-extrusion. (A) Micrographs of the water-in-oil single emulsion droplets; (B) Micrographs of the water-in-oil-in-water double emulsion droplets; (C) the effects of secondary emulsification times towards relative droplet number (▴) and mean fluorescence intensity (•) (reflecting the internal water phase volume) in P1, the fluorescence intensity decreased slightly with the increase of emulsification times, suggesting that over emulsification would disrupt the inner water phase of droplets; (D) Fluorescence peaks of w/o/w double emulsions incubated for different periods of time at room temperature: (red) 0 h, (green) 6 h, (blue) 22 h.

The water-in-oil (w/o) single emulsion droplets were generated by dispersing the inner water phase into oil phase through a Track-Etch polycarbonate membrane equipped in a mini-extruder. The diameter of the droplets decreased with the increase of emulsification times. There was no obvious difference in diameter whether to use 8-µm-pored or 12-µm-pored membrane. After optimization, we found that the emulsification times should be between 15.5 and 25.5 using 8-µm-pored membrane (Fig. 1A and Fig. S1 in Data S1). Under this condition, w/o single emulsion droplets can be reproducibly generated with diameters around 3∼5 µm, which are suitable for encapsulating *E. coli* cells with diameters of 1∼3 µm.

The water-in-oil-in-water (w/o/w) double emulsion droplets were generated by dispersing the primary single emulsion into outer water phase through 8-µm-pored membrane. To monitor the formation of emulsion droplets by flow cytometry, 100 µM *7-Hydroxycoumarin-3-carboxylic acid* was added to the inner water phase as a fluorescence marker. The droplets with reasonable volume of the inner water phase were gated as Research Population P1 by evaluating the fluorescence intensity (DAPI), the forward scatter (FSC) and the side scatter (SSC) values (Fig. S2 in Data S2). All the further researches regarding the micro-reactors were only focus on this P1 population. With the proceeding of the secondary emulsification, the number of w/o/w double emulsion droplets increased and their diameters kept decreasing until closed to the size of the pore on the membrane (∼8 µm) (Fig. S3 and Fig. S4 in Data S3). Further increasing the emulsification times could not increase the yield of droplets in P1 but would break the inner water phase slightly (Fig. 1C). Thus, the optimal secondary emulsification operation was chosen to be 25.5∼30.5 times (Fig. 1B, C). The w/o/w double emulsion droplets generated this way were ∼10 µm in diameter and compatible with flow cytometry. There was no significant change both in the diameter and the fluorescence intensity of the droplets after they were incubated at room temperature for 22 h (Fig. 1D). The good stability would be beneficial for the subsequent enzymatic reaction and FACS screening.

### Comparison of Droplet Uniformity between Membrane-extrusion and Homogenizing Method

**Figure 2:**
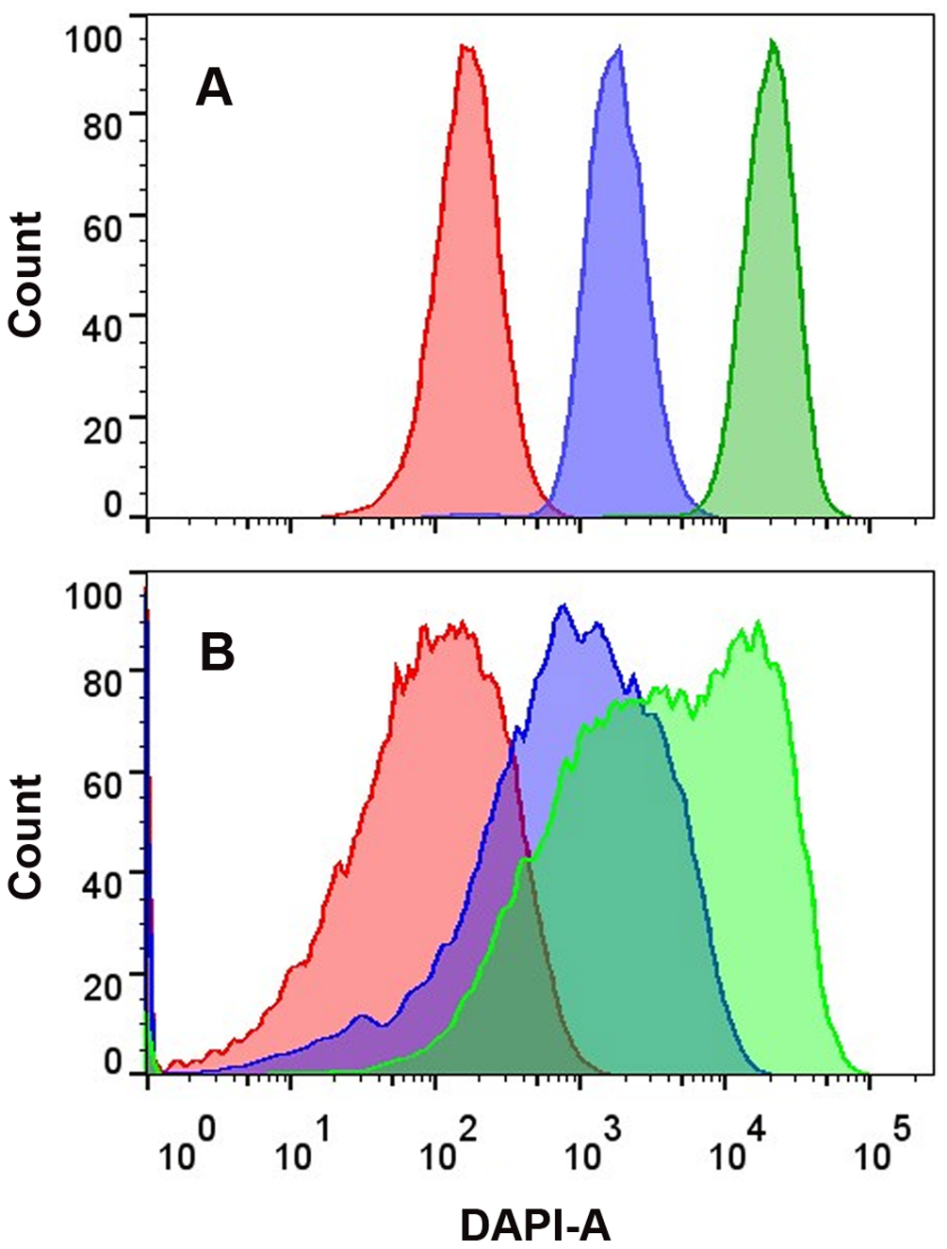
Comparison of volume uniformity of the internal water phase of w/o/w double emulsion droplets generated by membrane-extrusion (A) and homogenizing (B). Different-colored peaks represent different concentrations of *7-Hydroxycoumarin-3-carboxylic acid* in the internal water phase: (red) 10 µM, (blue) 100 µM, (green) 1000 µM.

To compare the performance of membrane-extrusion method with the most commonly used homogenizing method, w/o/w double emulsions were also prepared by homogenizing according to the published protocol [3]. The droplets generated by these two approaches had similar size under microscope and showed similar FSC-SSC dot plots in flow cytometry (Fig. S5 in Data S4). However, when *7-Hydroxycoumarin-3-carboxylic acid* was added into the inner water phase of the emulsions, it was found that the droplets generated by membrane-extrusion showed much narrower fluorescence peaks than that generated by homogenizing (Fig. 2). This phenomenon revealed that membrane-extrusion could generate droplets possessing much higher uniformity in inner water phase than that of the homogenizing method. The fluorescence peaks of homogenizing droplets were very wide, implying significant diversity in the inner water phase volume. In fact, this result is very similar to the other published work [10], which account for the large system error of current IVC-FACS format. The polydispersity might result from the strong shear stress of homogenizing process (with the rotor speed as high as 8000 rpm), which would break the w/o emulsion droplets during the secondary emulsification. In contrast, the membrane-extrusion method is much gentler and has less shear stress, which retains the integrity of the w/o emulsion and results in more homogeneous inner water phase in the w/o/w double emulsion. The outstanding uniformity of membrane-extrusion droplets is beneficial for accurate quantitation of enzymatic activity and promising better screening efficiency.

### Double Emulsion Droplets as Micro Enzymatic Reactor

**Figure 3:**
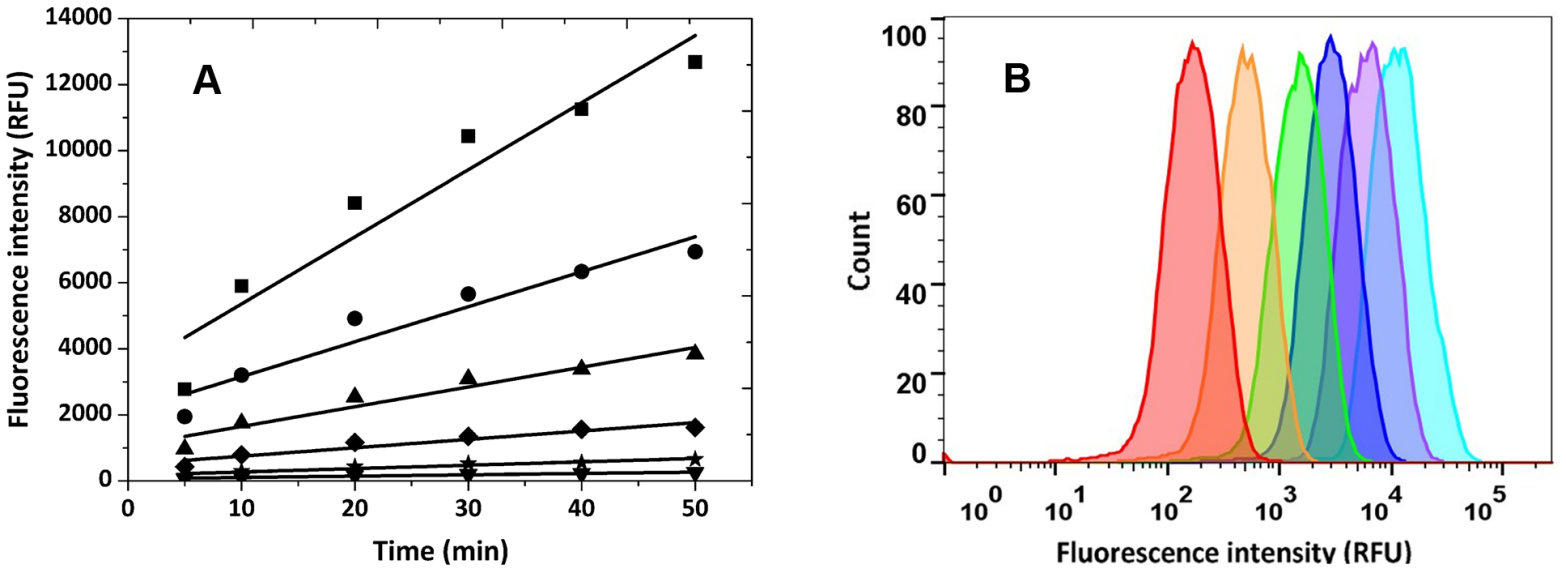
Free esterase reaction in w/o/w double emulsion droplets at 37°C. (A) Time courses of reactions under different concentrations of esterase AFEST (diluted folds of cell lysate): (▪) 1 fold, (•) 2 folds, (▴) 4 folds, (⧫) 8 folds, (★)16 folds, (▾) 32 folds; the slopes for the curves are 203.2(▪), 106.0(•), 59. 9(▴), 25.4(?), 10.2(★), and 4.0(▾). (B) Fluorescent signals of emulsions containing different dilutions of esterase AFEST (diluted folds of cell lysate) after reaction for 30 min: 1 fold (light blue), 2 folds (purple), 4 folds (dark blue), 8 folds (green), 16 folds (orange), 32 folds (red).

Before single cells were compartmentalized in the droplets, the kinetic behavior of free enzyme in these micro-reactors was investigated. We chose a thermophilic esterase from *Archaeoglobus fulgidus* (AFEST) as the model. Identified by Manco et al. in 2000 [15], AFEST shows good hydrolysis capability towards short chain fatty acid (C4∼C6) esters. It has an optimal temperature of ∼85°C, and is extremely stable at high temperatures. In this work, AFEST was over expressed in *E. coli* and the crude cell lysate was diluted at a 2-fold gradient, giving a series of enzyme solutions with the activity ratios of 1, 2, 4, 8, 16 and 32, respectively. These enzyme solutions were used as inner water phase and encapsulated into w/o/w double emulsions. Instead of encapsulating the substrate together with the enzyme into the droplets, 0.5 mM of fluorogenic substrate fluorescein dibutyrate was added in the outer water phase to decrease the fluorescence background which is caused by the unwanted reaction before droplet formation. The high hydrophobicity of the substrate allowed it pass through the oil phase very quickly, ensuring that the substrate permeation was not a rate-limiting step. The emulsions were incubated at 37°C for different periods of time and the fluorescence signals of the emulsions were analyzed by a flow cytometer. As shown in Fig. 3A, the average fluorescent intensity of the droplets showed excellent kinetic linearity at all the enzyme concentrations tested. This proved that the enzymatic reactions had very good dynamic range in the droplets. In addition, the reaction rate increased proportionally with the increasing of enzyme concentration, suggesting a good correlation of the kinetic behavior in the droplets with that in bulk reaction. The fluorescence peaks of different enzyme concentrations could be separated very well from each other at any reaction time measured (Fig. 3B, showing the reaction at 30 min as an example). To the best of our knowledge, this is the highest resolution that ever has been reported in such IVC-FACS systems. The high sensitivity should benefit from the improved uniformity of membrane-extrusion micro-reactors and is crucial for the high efficacy of the following screening.

### Single-cell Micro-reactors for IVC-FACS Assays of Esterases

**Figure 4:**
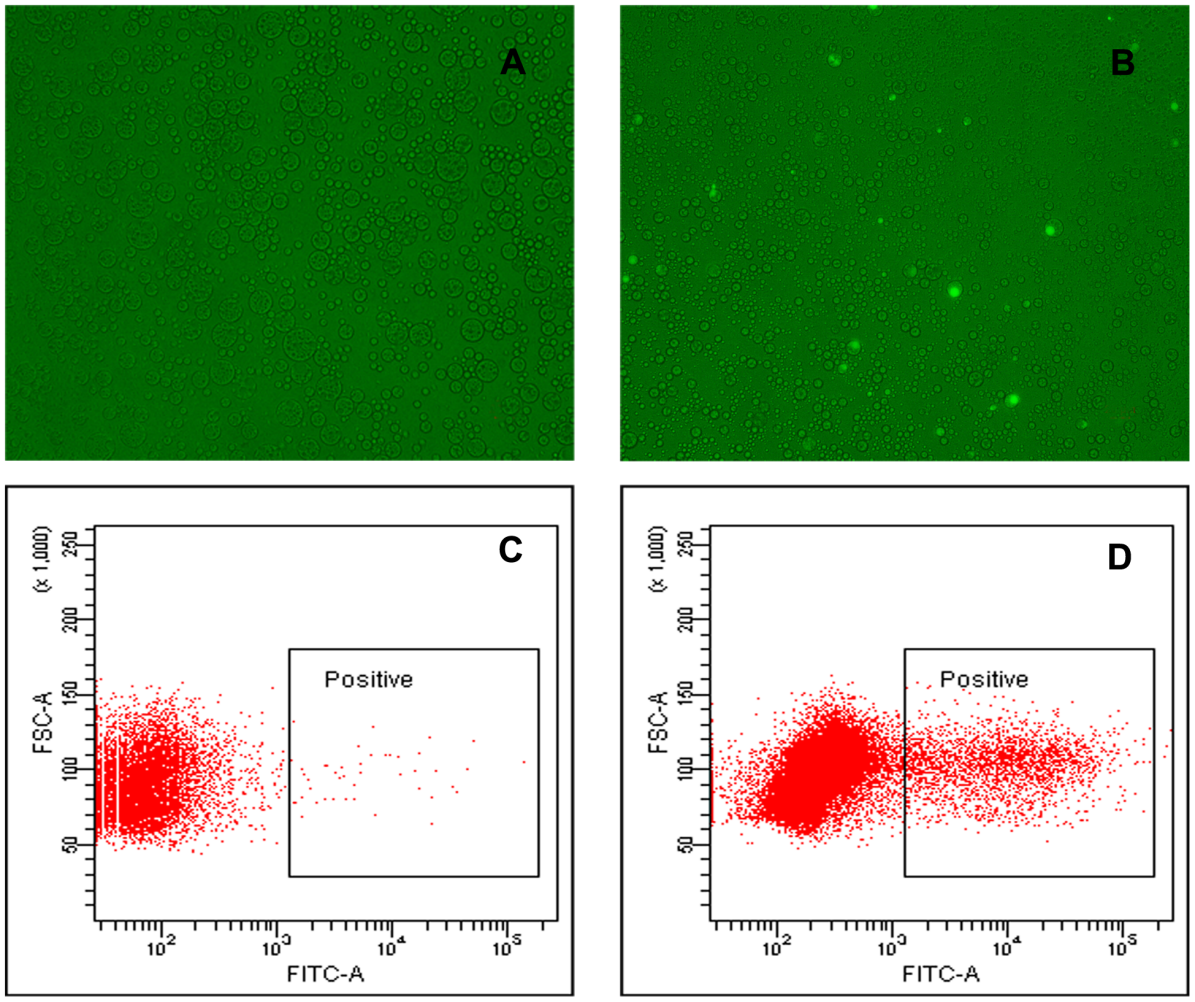
Fluorescence signal of single-cell enzymatic reaction in w/o/w double emulsion droplets. (A) Fluorescence micrograph of w/o/w double emulsion encapsulating *E. coli* JM109 cells harboring null pGF101 plasmid (negative cells) (40×object). (B) Fluorescence micrograph of w/o/w double emulsion encapsulating AFEST-displaying cells (40×object). (C) Dot-plot of the product fluorescence intensity of the w/o/w double emulsion encapsulating negative cells. (D) Dot-plot of the product fluorescence intensity of the w/o/w double emulsion encapsulating AFEST-displaying cells.

The thermophilic esterase AFEST was displayed on the surface of *E. coli* JM109 strain using the N- and C- terminal recombinant fragment of ice nucleation protein (INPNC) as the cell surface display carrier. This surface-displaying expression would facilitate the direct interaction between the substrate and enzyme. The AFEST-displaying *E. coli* JM109 cells and cells harboring null pGF101 plasmid were compartmentalized into w/o/w double emulsion droplets, respectively. After an optimization, the concentration of cells was controlled at ∼2.5×10^8^/100 µL to balance a higher throughput and a lower ratio of the multi-cell-containing droplets (see Fig. S6 in Data S5 for details). Fluorescein dibutyrate was added to the outer water phase to a final concentration of 0.5 mM and the emulsions were incubated at 37°C for 30 min. After reaction, the fluorescence of the emulsion was detected by fluorescence microscope and flow cytometry (Fig. 4). Significant fluorescence signal in AFEST-encapsulating emulsion was observed by fluorescence microscope (Fig. 4B). The brighter spots represented cell-containing droplets with higher fluorescence, while the darker ones were the null droplets. Similar fluorescence distinction was also observed by flow cytometry (Fig. 4D). The emulsion droplets containing the cells (∼10% of all) and null ones (∼90% of all) were divided into two populations with different fluorescent intensity. On the contrary, the fluorescence signal of droplets encapsulating esterase-free *E. coli* JM109 cells was too low to be distinguished from that of the null droplets neither by the fluorescence microscope nor by the flow cytometry (Fig. 4A, C).

**Figure 5:**
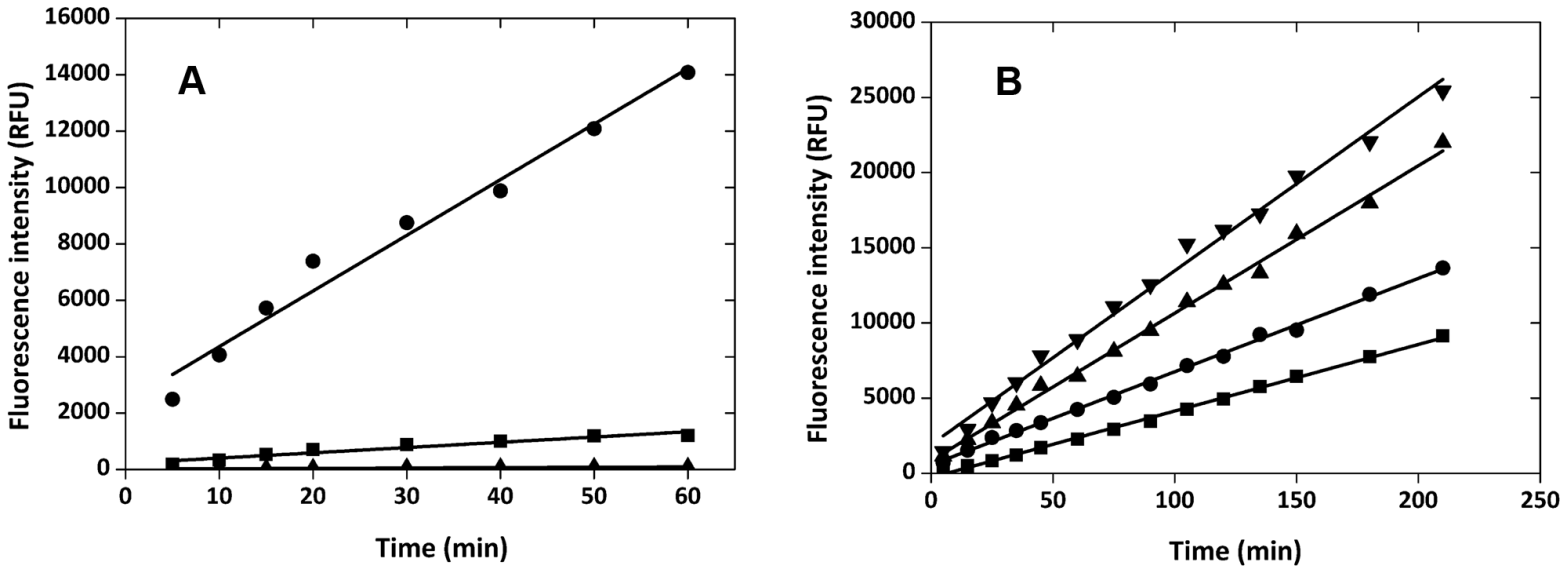
Enzymatic reactions in w/o/w double emulsion droplets encapsulating single cells. (A) Reactions of AFEST-displaying cells and FnE-displaying cells encapsulating droplets with 0.5 mM fluorescein dibutyrate: (▴) null droplets in AFEST-encapsulating sample, (▪) FnE-containing droplets, (•) AFEST-containing droplets. (B) Reactions of AFEST-displaying cells in droplets with different fluorescein dibutyrate concentrations: (▪) 0.5 mM, (•) 2 mM, (▴) 5 mM, (▾) 10 mM.

To further test the sensitivity of this single cell IVC-FACS system, another thermophilic esterase, FnE from *Fervidobacterium nodosum,* was introduced as a secondary model enzyme. The activity of FnE was about 8-fold lower than that of AFEST when displayed on the surface of *E. coli* JM109. The cells displaying AFEST or FnE on their surface were encapsulated into w/o/w double emulsions for reaction, respectively. The time courses of reaction were showed in Fig. 5A. The reaction rate of AFEST was much higher than that of FnE, with a difference about 11-fold, which was comparable with that of in bulk reaction. These results confirmed that single *E. coli* cells with different enzymatic activities could be distinguished very well in the w/o/w micro-reactors generated by our protocol.

The single-cell reactions inside the micro-reactors were further investigated with different substrate concentrations. W/o/w double emulsions encapsulating AFEST-displaying cells were incubated with 0.5–10 mM of fluorescein dibutyrate at 37°C, and the fluorescent intensity of the emulsions were measured by flow cytometry over time. As shown in Fig. 5B, reaction rate increased along with the increasing of substrate concentrations. The fluorescence intensity and reaction time showed good linearity over all the substrate concentrations tested. The apparent *K*_M_ value of AFEST for fluorescein dibutyrate could be estimated by the relative rate of fluorescence increase. The result turned out to be 700 µM, which was comparable with the value obtained from a 0.8 mL quartz cuvette by a fluorescence spectrophotometry (240 µM, Fig. S7 in Data S6). This result again confirmed that the kinetic behavior in the emulsion droplets was very similar to that in a bulk reaction system, and was also a strong proof of the high accuracy of our IVC-FACS system.

### Validation of the IVC-FACS System by Model Screening

Model screening was performed to evaluate the utility of the IVC-FACS system. AFEST-displaying cells were mixed with cells harboring null pGF101 plasmid at 1∶10, 1∶100, and 1∶1000 ratios, respectively. The cell mixtures were then encapsulated into w/o/w double emulsion droplets for reaction. Different percentages (0.1% and 0.5%) of the droplets with the highest fluorescence were collected and plated on LB agar plates. The presence of AFEST activity in the resulting colonies was identified in 96-well plates using 4-nitrophenyl butyrate as substrate. The enrichment factors were calculated according to the percentages of the positive cells before and after sorting. As shown in Table 1, the positive colonies could be enriched significantly after only one round of sorting. Even when the positive ratio at beginning was as low as 0.1%,a single round of high-stringency sorting could bring the ratio up to 33%, with an enrichment factor of 330 folds. This model screening clearly demonstrated that AFEST-displaying cells could be sorted and enriched from a large excess of non-active cells with very high efficiency even at very low positive ratios.

**Table 1:** Model screening of AFEST-displaying cells.

Before sorting Positive cells/all	Gating Percentage sorted ofbrightest events	After sorting Positive cells/all	Enrichment
1/10	0.5%	23/24	**10**
1/10	0.1%	21/24	**9**
1/100	0.5%	18/22	**82**
1/100	0.1%	22/24	**92**
1/1000	0.5%	4/24	**167**
1/1000	0.1%	8/24	**333**

### Directed Evolution of Thermophilic Esterase AFEST

The high accuracy of our IVC-FACS system is a valuable feature, especially in the case of directed evolution of industrial enzymes. Most industrial enzymes are already very efficient after iterative rounds of engineering and evolution. The evolution space became limited when the *k*_cat_/*K*_M_ approaching the diffusion-limited efficiency (∼10^8^ M^−1^s^−1^), and it is challenging to further improve their catalytic activities. Even though, small improvements is also important given the huge market output value of many industrial enzymes. Therefore, high-throughput screening method with accurate mutant activity readout is strongly desired. As stated above, our IVC-FACS system combines the power of ultrahigh-throughput and high sensitivity, which holds great potential to identify the mutants with small improvement from an already very efficient parent enzyme. To validate this, AFEST was chosen as a model for a directed evolution experiment. AFEST is a very effective esterase towards the short chain fatty acid esters. Its catalytic efficiency (*k*_cat_/*K*_M_) for 4-nitrophenyl butyrate is ∼6×10^6^ M^−1^s^−1^ at 37°C, only ∼16 times slower than the diffusion-limited efficiency. The high activity of AFEST makes it’s a challenging task to further improve its catalytic efficiency, and is a strict test for our IVC-FACS system.

The random mutagenesis library of AFEST (2–5 mutations per gene, containing ∼2×10^6^ mutants) was constructed by error-prone PCR. The library was expressed in *E. coli* JM109, and the single cells were encapsulated into w/o/w double emulsion for screening. About 0.1% droplets with the highest fluorescent intensity were sorted into 2-mL eppendorf tubes and the positive genes were directly amplified from the sorted cells using PCR. The genes were recloned into pET-28a(+) plasmid and transformed into *E. coli* BL21-CodonPlus (DE3). About 500 clones were picked into 96 well plates for the secondary screening. Nineteen mutants showing higher activity towards 4-nitrophenyl butyrate over wild type AFEST were obtained (Table S1 in Data S7), and the highest three mutants were identified to be V293E, V293G and S94G/F287Y (Table 2).

**Table 2:** Kinetic parameters of the wild type AFEST and its mutants.^a.^

Variant	*K*_M_ (µM)	*k*_cat_ (s^−1^)	*k*_cat_/*K*_M_ (×10^6^ M^−1^s^−1^)
WT	22.47	135.49	6.03
V293E	12.13	138.15	11.38
V293G	9.74	117.93	12.10
S94G/F287Y	15.42	154.54	10.02

a Kinetic parameters were measured as 37°C, using 4-nitrophenyl butyrate as substrate.

The sensitivity of the IVC-FACS system was validated again by a model screening of the V293E mutant out of the wide-type AFEST. The *E. coli* cells expressing V293E could be enriched from the cells expressing wide-type AFEST, suggesting that 2-fold changes in catalytic activity is enough to be separated by this IVC-FACS system (see Fig. S8 in Data S8 for detailed information).

The *k*_cat_/*K*_M_ value of the best mutant V293G was ∼1.2×10^7^ M^−1^s^−1^ (Table 2), approximately 2-times more active than the wild type enzyme, and only 8-fold lower than the diffusion-limited efficiency of ∼10^8^ M^−1^s^−1^. Further kinetic analysis suggested that the activity increase was mainly caused by the reduced *K*_M_ value, while there were no significant changes in *k*_cat_ value (Table 2). Because the substrate concentration in the screening (0.5 mM) was lower than the apparent *K*_M_ of AFEST in droplets (∼0.7 mM), mutants with decreased *K*_M_ value would show higher catalytic efficiency and thus be identified. This result was consistent with the first law of directed evolution “you get what you screen for”. Interestingly, the mutants retained the excellent thermostablity of the wild type enzyme. The optimal temperature of the improved mutants was similar to that of the wild type AFEST (Fig. S9 in Data S9), suggesting there was no trade-off between activity and thermostability during the evolution process. This makes the mutants to have higher catalytic activity in the entire temperature range compared with the wild type enzyme, and have the potential to be better biocatalysts for industry applications.

**Figure 6:**
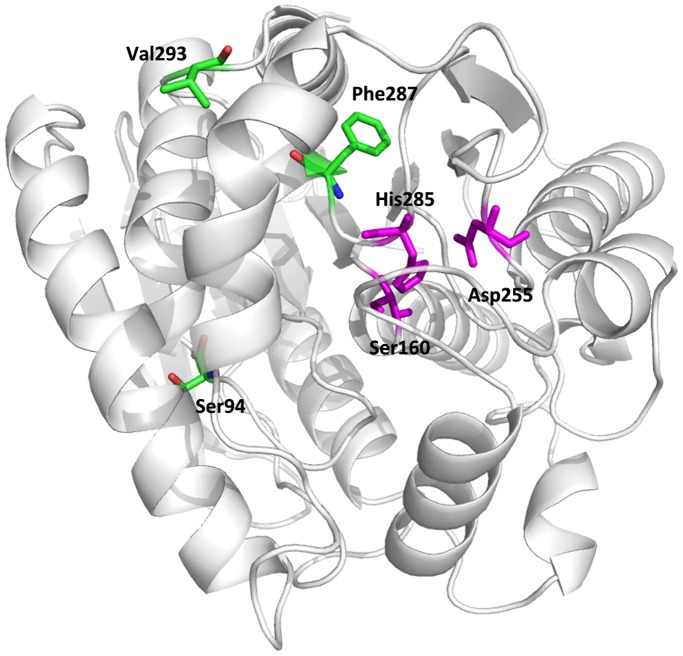
Positions of the positive mutation sites in AFEST. Mutated residues are marked in green and catalytic residues of AFEST are marked in purple.

According to the crystal structure of AFEST, all the positive mutation sites occurred far from the active site (Fig. 6), suggesting the effect of higher substrate binding affinity might be a consequence of remote interactions. This kind of mutations are very difficult to be found by regular rational design approaches, which highlight the importance of directed evolution in the optimization of catalytic activities. Because the wild type AFEST is already a very efficient enzyme, the beneficial mutations for the catalytic activity should be relative rare and require the searching of larger sequence space to be found. With the help of this new IVC-FACS system, we were able to identify several beneficial mutations from 2×10^6^ variants of AFEST with relative high mutation rate (2–5 mutations per gene), which again proved the efficiency and sensitivity of our IVC-FACS system.

## Conclusion

The generation of w/o/w droplets is one of the most critical steps in an IVC-FACS screening system: the uniformity of the w/o/w droplets is the key for the screening accuracy, while the speed of droplet generation is important for the throughput. Homogenizing is currently the most commonly used method for droplet generation, but the wide polydispersity of the micro-reactors severely limits its accuracy [3]. Microfluidics, on the other hand, showed the ability to produce w/o/w droplets with extremely uniform diameters, but it is still difficult to generate very small w/o/w droplets (less than 1/3 of the 70 µm nozzle of the cytometer) at ultrahigh speed to fulfill the highest sorting efficiency of FACS [12]. The method described in this article provides higher accuracy than the homogenizing method and faster speed for the w/o/w droplets generation than the microfluidics (Table 3). Importantly, it provides enough screening accuracy for most of the applications and is very easy to handle. In this perspectives, it has the potential to be a more feasible ultrahigh-throughput screening method before the final mature of the microfluidics system, thereby facilitates the discovery and engineering of important industrial enzymes.

**Table 3:** Comparison among three different w/o/w double emulsion droplet generation methods.

W/o/w droplet generation method	Technicalrequirement	Droplet diameter	Generating speed	Uniformity	References
Homogenizer	Low	∼10 µm	∼10^10^/10 min	Poor	[3]
Microfluidic chip	High	>50 µm	<10^6^/10 min	High	[12]
Mini extruder	Low	∼10 µm	∼10^10^/10 min	Medium	This work

## Supporting Information

Data S1 **W/o single emulsion droplets generated by primary emulsification. (Fig. S1)**
(DOCX)

Data S2 **Selection of Research Population P1. (Fig. S2)**
(DOCX)

Data S3 **W/o/w double emulsion droplets generated by secondary emulsification. (Fig. S3 and Fig. S4)**
(DOCX)

Data S4 **Comparison of the double emulsion droplets generated by the homogenizing method and the membrane-extrusion method. (Fig. S5)**
(DOCX)

Data S5 **Optimization of the cell concentration by double-color encapsulation. (Fig. S6)**
(DOCX)

Data S6 **Comparison of the kinetic behavior of AFEST in bulk reaction and in the micro-reactors. (Fig. S7)**
(DOCX)

Data S7 **Positive mutants obtained from the secondary screening. (Table S1)**
(DOCX)

Data S8 **Model screening of wide-type AFEST and its V293E mutant. (Fig. S8)**
(DOCX)

Data S9 **Optimal temperatures of AFEST and its mutants. (Fig. S9)**
(DOCX)
