# Supplementary material for: An Improved Single Cell Ultrahigh Throughput Screening Method Based on In Vitro Compartmentalization
Source: PLoS One. 2014 Feb 24;9(2):e89785. doi: 10.1371/journal.pone.0089785 (PMC3933655; doi:10.1371/journal.pone.0089785)
Supplement: Data S1 — W/o single emulsion droplets generated by primary emulsification. (Fig. S1) (DOCX) [file pone.0089785.s001.docx]

**S1. W/o single emulsion droplets generated by primary emulsification.**

For the primary emulsification, the internal water phase was dispersed into oil phase by passing the 8-µm pores in the polycarbonate membrane. The diameter of the droplets was measured by a microscope at the end of different emulsification times (7.5, 15.5, 20.5 and 30.5). As shown in Fig S1, the diameter of dispersed internal water phase decreased with the increase of emulsification times. The optimal emulsification time was between 15.5 and 25.5, in which case the majority of droplets were 3~5 µm. With the emulsification time less than 7.5 or exceeding 35.5, the droplet size was either too large (>10 µm) or too tiny (<2 µm).

**
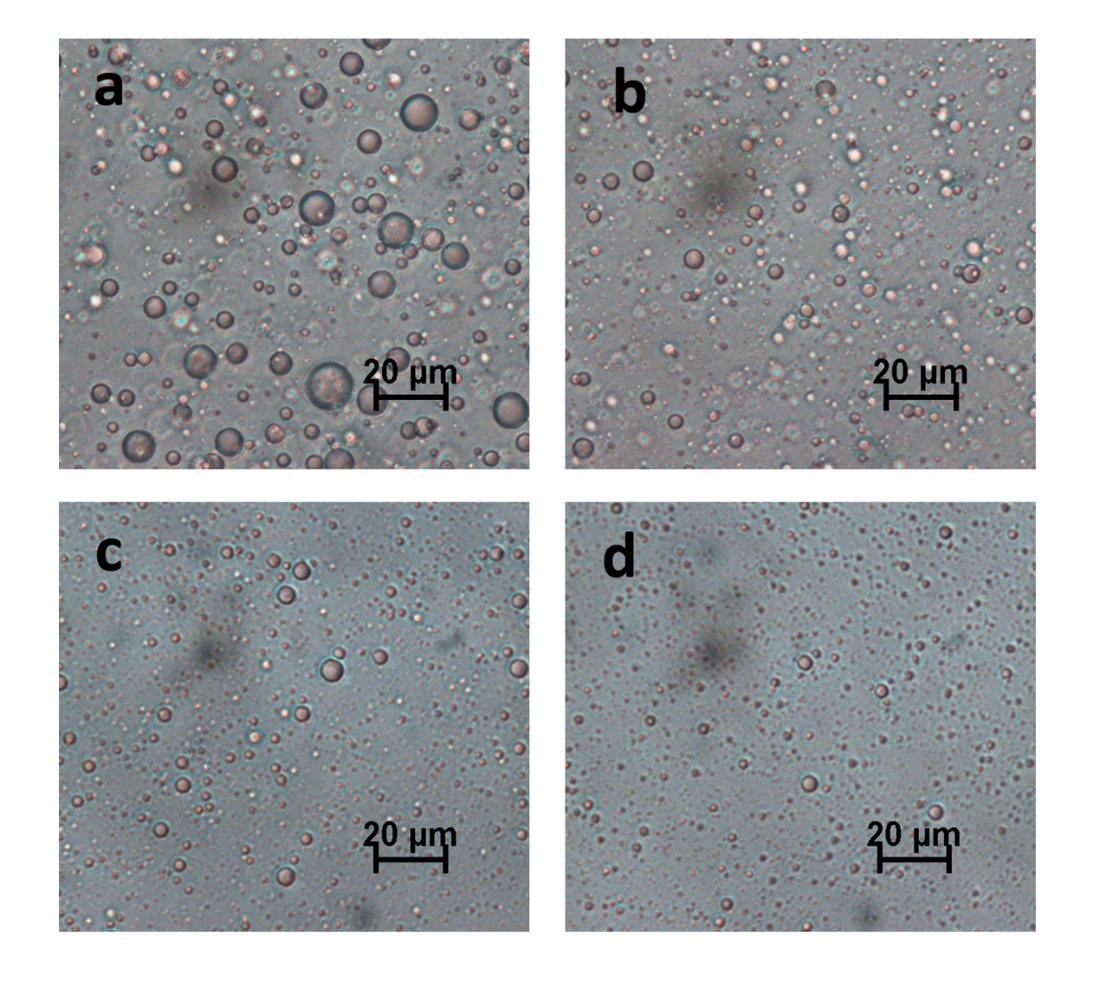
**

**Fig. S1** Micrographs of single emulsion droplets generated by various emulsification times. (a. 7.5 times, b. 15.5 times, c. 20.5 times, and d. 30.5times).
