# Supplementary material for: An Improved Single Cell Ultrahigh Throughput Screening Method Based on In Vitro Compartmentalization
Source: PLoS One. 2014 Feb 24;9(2):e89785. doi: 10.1371/journal.pone.0089785 (PMC3933655; doi:10.1371/journal.pone.0089785)
Supplement: Data S2 — Selection of Research Population P1. (Fig. S2) (DOCX) [file pone.0089785.s002.docx]

**S2. Selection of Research Population P1.**

By adding 100 µM 7-Hydroxycoumarin-3-carboxylic acid as a fluorescence marker, the volume of inner water phase could be evaluated by the fluorescence intensity of the droplets (DAPI). Meantime, the diameter and the inner complexity of a droplet could be reflected by Forward Scatter (FSC) and Side Scatter (SSC) values, respectively. As shown in Fig. S2a and Fig. S2b, the DAPI values of the droplets had a correlation with the FSC and the SSC values, respectively. Therefore, the droplets with medium fluorescence intensity (DAPI) were chosen by using the corresponding value of FCS and SSC (50<FSC<150 and 50<SSC<150) as criteria. These droplets were defined as Research Population P1 (Fig. S2c), which were considered having a reasonable volume of the inner water phase. P1 population also excluded the droplets with too large or too small inner water phase, presenting the micro-reactors with a reasonable quality for further researches.


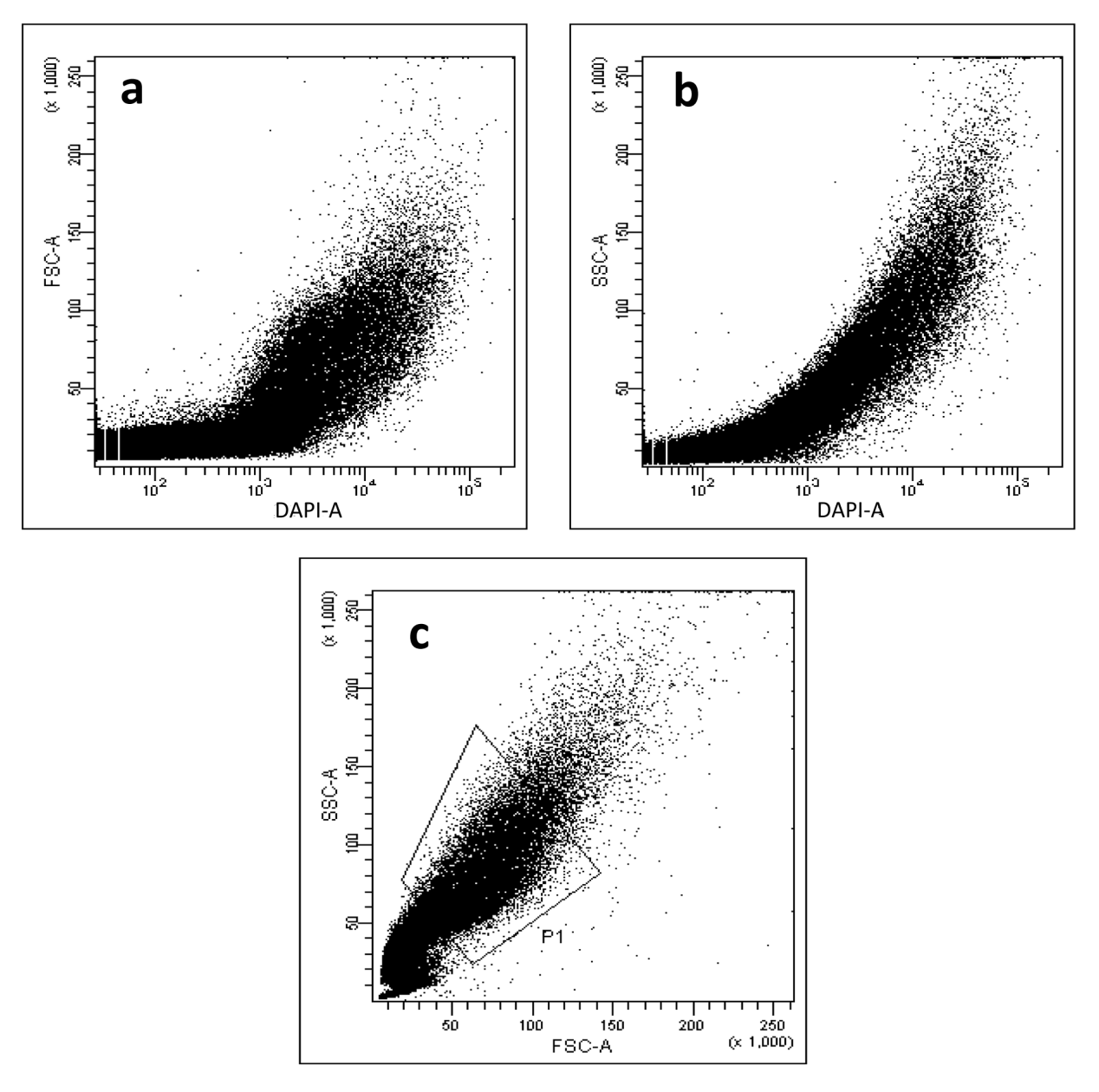


**Fig. S2.** Parameters of w/o/w double emulsion droplets measured by flow cytometry. (a) DAPI-FSC dot plot; (b) DAPI-SSC dot plot; (c) FSC-SSC dot plot and research population P1 selection.
