# Supplementary material for: An Improved Single Cell Ultrahigh Throughput Screening Method Based on In Vitro Compartmentalization
Source: PLoS One. 2014 Feb 24;9(2):e89785. doi: 10.1371/journal.pone.0089785 (PMC3933655; doi:10.1371/journal.pone.0089785)
Supplement: Data S3 — W/o/w double emulsion droplets generated by secondary emulsification. (Fig. S3 and Fig. S4) (DOCX) [file pone.0089785.s003.docx]

**S3. W/o/w double emulsion droplets generated by secondary emulsification.**

W/o/w double emulsion was generated by dispersing the primary w/o single emulsion into outer water phase through an 8-µm-pored membrane. With the increasing of emulsification times, the w/o single emulsion was gradually divided into smaller fractions to form w/o/w double emulsion. When the emulsification times exceeding 25.5, there was no significant change in droplet size (Fig. S3), and the size distribution of the droplets remained stable in flow cytometry (Fig. S4). The final droplet diameter remained within ~10 µm, which was comparable with the pore size of the membrane (8 µm).

**
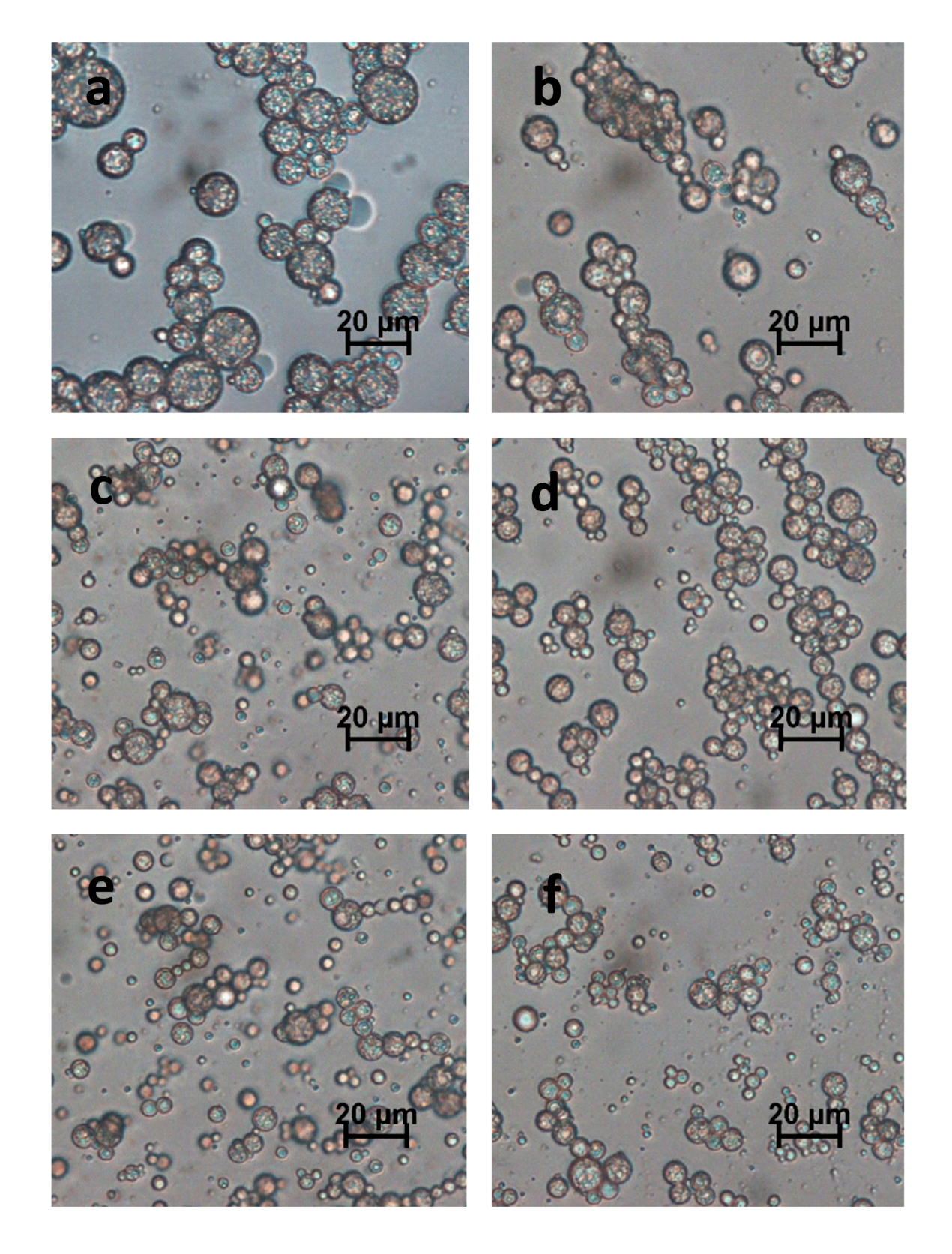
**

**Fig. S3** Micrographs of double emulsion droplets generated by various secondary emulsification times.

**

**

**Fig. S4.** FSC-SSC dot plots of double emulsion droplets generated by various secondary emulsification times.
