# Supplementary material for: An Improved Single Cell Ultrahigh Throughput Screening Method Based on In Vitro Compartmentalization
Source: PLoS One. 2014 Feb 24;9(2):e89785. doi: 10.1371/journal.pone.0089785 (PMC3933655; doi:10.1371/journal.pone.0089785)
Supplement: Data S4 — Comparison of the double emulsion droplets generated by the homogenizing method and the membrane-extrusion method. (Fig. S5) (DOCX) [file pone.0089785.s004.docx]

**S4. Comparison of the double emulsion droplets generated by the homogenizing method and the membrane-extrusion method.**

The membrane-extrusion method was done as described in the main text. The homogenizing method was performed as described by Aharoni et al. (2005). Briefly, 80 µL PBS buffer (pH7.4) containing 100 µM *7-Hydroxycoumarin-3-carboxylic acid* was used as internal water phase. It was added to a 2 mL round-bottom cryotube (Corning) which containing 800 µL oil phase (light mineral oil containing 2.9% ABIL EM90). The mixture was homogenized for 5 min at 9500 rpm on ice using the OMNI THQ homogenizer equipped with a blunt tip, making a primary emulsion. Then 800 µL secondary water phase (pH7.4 PBS containing 1% TritonX-102 and 1.5 medium viscosity CMC) was added, and the mixture was homogenized at 8000 rpm for 3 min to give the w/o/w double emulsion. The droplets generated by the two methods was loaded on a FACSAria^TM^ II cytometer. They showed comparable proprieties in the FSC-SSC dot plots (Fig. S5).

**

**

**Fig. S5.** FSC-SSC dot plots of double emulsion droplets generated by homogenizing method (a) and membrane-extrusion method (b).
