# Supplementary material for: An Improved Single Cell Ultrahigh Throughput Screening Method Based on In Vitro Compartmentalization
Source: PLoS One. 2014 Feb 24;9(2):e89785. doi: 10.1371/journal.pone.0089785 (PMC3933655; doi:10.1371/journal.pone.0089785)
Supplement: Data S5 — Optimization of the cell concentration by double-color encapsulation. (Fig. S6) (DOCX) [file pone.0089785.s005.docx]

**S5. Optimization of the cell concentration by double-color encapsulation**

As a single-cell enzyme screening system, the proportion of multi-cell-containing droplets should be kept at a low level to avoid potential false positive results. However, it’s also important to make the cell-containing droplets as many as possible to give a high throughput. Therefore, it is crucial to find a best concentration of the cells for the compartmentalization.

Two kinds of *E. coli* cells expressing mCherry fluorescence protein (mCherry) and green fluorescence protein (GFPuv), respectively, were mixed at a ratio of 1:1 and were encapsulated into w/o/w droplets together. The two colors of fluorescence allow us to calculate the percentage of multi-cell containing droplets as a function of cell concentration. As shown in Fig. S6a, the population Q2 presents the droplets encapsulating both mCherry-expressing cells and GFPuv-expressing cells. The total number of multi-cell containing droplets was estimated as 3 times of Q2 (namely, mCherry-GFPuv, mCherry-mCherry and GFPuv-GFPuv). Therefore, the percentage of multi-cell containing droplets could be calculated using the following equation:

$$Mutil\%=\frac{3\times Q2}{Q1+Q2+Q4}$$

As shown in Fig. S6c, the percentage of multi-cell droplets increases dramatically with the increase of cell concentration. Considering both higher throughput and lower ratio of multi-cell droplets, we choose the cell concentration of 2.5×10^8^/100 µL, in which case the percentage of multi-cell droplets in all the cell-containing droplets is ~8%.


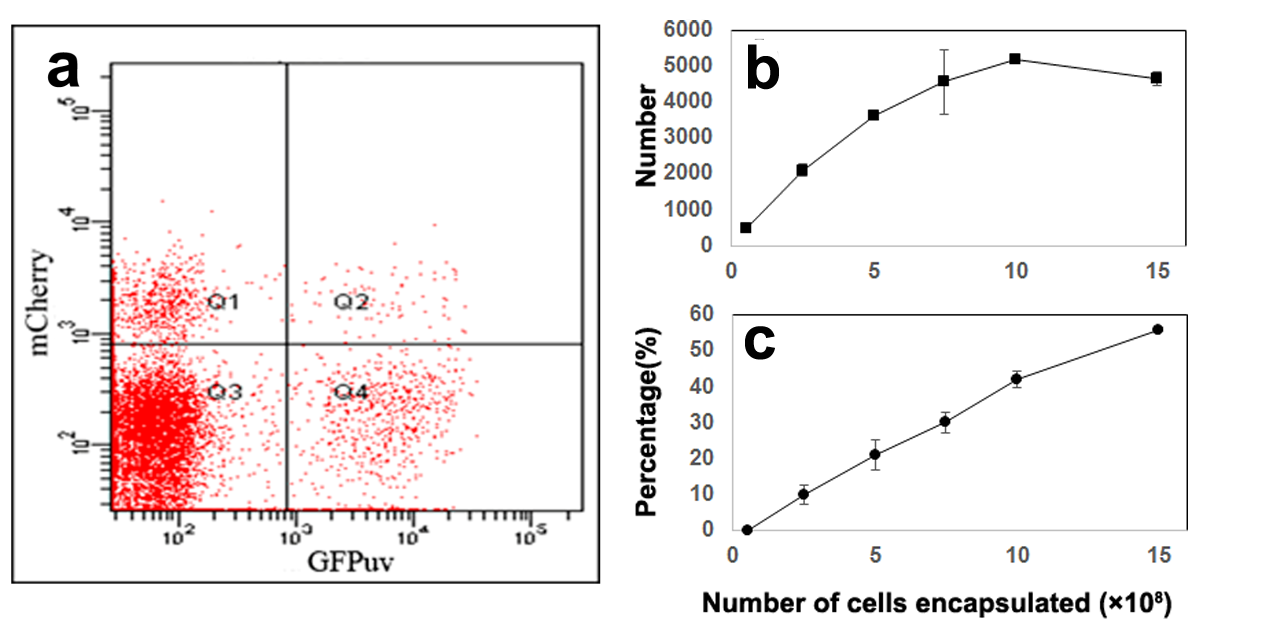


**Fig. S6.** Double-color encapsulation assays for measuring percentage of multi-cell droplets. a) dot-plot of droplets encapsulating mixture of GFPuv-expressing cells and mCherry-expressing cells; b) number of all the cell-containing droplets (■) in P1 population (30,000 events in total) as a function of cell concentration; c) the percentage of multi-cell droplets (●) in all the cell-containing droplets as a function of cell concentration. The results in Fig. S6b and Fig. S6c were the average of three experiments.
