# Supplementary material for: An Improved Single Cell Ultrahigh Throughput Screening Method Based on In Vitro Compartmentalization
Source: PLoS One. 2014 Feb 24;9(2):e89785. doi: 10.1371/journal.pone.0089785 (PMC3933655; doi:10.1371/journal.pone.0089785)
Supplement: Data S6 — Comparison of the kinetic behavior of AFEST in bulk reaction and in the micro-reactors. (Fig. S7) (DOCX) [file pone.0089785.s006.docx]

**S6. Comparison of the kinetic behavior of AFEST in the bulk reaction and in the micro-reactors.**

The relative reaction rates of AFEST against different concentrations of fluorescein dibutyrate were measured in bulk reaction and in the micro-reactors, respectively. In the bulk reaction, the purified AFEST was reacted with fluorescein dibutyrate at 37 ^o^C in PBS buffer. The release of fluorescein was measured by a Hitachi F-7000 Fluorescence Spectrophotometer. The slope of fluorescence increasing curve was regarded as the relative reaction rate. In the droplet micro-reactors, single *E. coli* cells displaying AFEST were encapsulated into w/o/w double emulsion droplets and then the droplets were incubated with different concentrations of fluorescein dibutyrate at 37 ^o^C. The average fluorescence of different reaction time was measure by a FACSAria^TM^ II flow cytometer. Time courses of reactions under each substrate concentration were obtained and the slope was calculated as the relative reaction rate.

The *K*_M_ values were calculated according to the double-reciprocal plot derived from Michaelis-Menten equation (v=V_max_S/(*K*_M_+S)). In the double-reciprocal plot, the value of x intercept represents -1/*K*_M_. *K*_M_ of AFEST to fluorescein dibutyrate in bulk and in droplets was calculated as 240 µM and 700 µM, respectively (Fig. S7). This result showed that the kinetic behavior in the emulsion droplets is very similar to that in a bulk reaction system.

**
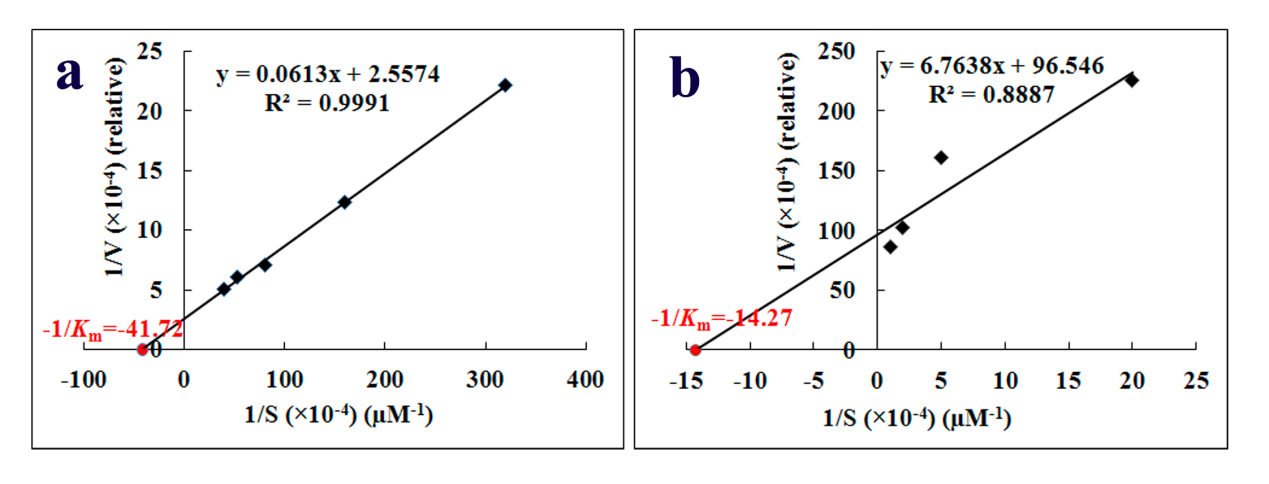
**

**Fig. S7.** *K*_M_ of AFEST to fluorescein dibutyrate in bulk (a) and in w/o/w droplets (b). Reaction rates were measured at 37 ^o^C.
