# Supplementary material for: An Improved Single Cell Ultrahigh Throughput Screening Method Based on In Vitro Compartmentalization
Source: PLoS One. 2014 Feb 24;9(2):e89785. doi: 10.1371/journal.pone.0089785 (PMC3933655; doi:10.1371/journal.pone.0089785)
Supplement: Data S7 — Positive mutants obtained from the secondary screening. (Table S1) (DOCX) [file pone.0089785.s007.docx]

**S7.** Positive mutants obtained from the secondary screening.

**Table S1.** Mutation sites of positive mutants obtained from the secondary screening.

| **Mutant name** | **Mutation modes** |
| --- | --- |
| 2D3 | Q49R |
| 3A2 | I237V |
| 11A4 | V293E |
| 5E8 | L47R, R103G, K295R |
| 8C8 | D299G |
| 8G11 | D58G, T137A, F287S |
| 4E4 | S94G |
| 3E4 | V293G |
| 5B5 | I237T |
| 7G9 | T183Y, N289D, L294P |
| 8B11 | S216G, L294P |
| 9B1 | R103G, S176G, I288T, V293E |
| 10D2 | S94N, S111T |
| 12B3 | N37S, L47P, S94G |
| 2H4 | S94G, F287Y |
| 1F1 | N192S, V293A |
| 1G10 | I39V, L201P, V293G |
| 2D3 | R38Q, K295N |
| 3D1 | Q49R, A166V, D175V, K295N |
