# Supplementary material for: An Improved Single Cell Ultrahigh Throughput Screening Method Based on In Vitro Compartmentalization
Source: PLoS One. 2014 Feb 24;9(2):e89785. doi: 10.1371/journal.pone.0089785 (PMC3933655; doi:10.1371/journal.pone.0089785)
Supplement: Data S8 — Model screening of wide-type AFEST and its V293E mutant. (Fig. S8) (DOCX) [file pone.0089785.s008.docx]

**S8. Model screening of wide-type AFEST and its V293E mutant.**

To further validate the sensitivity of our IVC-FACS system, the *E. coli* JM109 cells displaying wide-type AFEST and mutant V293E (about 2-fold as active as WT) were mixed together at a ratio of 1:1. The cell mixture was encapsulated and reacted with fluorescein dibutyrate. About 0.1% droplets with the highest fluorescent signal were sorted and plated on agar plate. Twenty clones were sequenced and the results showed that 18 of them were identified to be V293E. The ratio of V293E was improved from 50% to 90% after one round of FACS, suggesting that 2-fold changes in catalytic activity is enough to be separated by this IVC-FACS system.**

**

**Fig. S8.** Model screening of wide-type AFEST and a 2-fold improved mutant V293E.
