# Supplementary material for: An Improved Single Cell Ultrahigh Throughput Screening Method Based on In Vitro Compartmentalization
Source: PLoS One. 2014 Feb 24;9(2):e89785. doi: 10.1371/journal.pone.0089785 (PMC3933655; doi:10.1371/journal.pone.0089785)
Supplement: Data S9 — Optimal temperatures of AFEST and its mutants. (Fig. S9) (DOCX) [file pone.0089785.s009.docx]

**S9. Optimal temperatures of AFEST and its mutants.**

The improved mutants showed similar optimal temperature with that of the wild type AFEST (about 85 ^o^C).





**Fig. S9.** Optimum temperature of AFEST and its mutants.
